# Influence of Surface Defects and Size on Photochemical Properties of SnO_2_ Nanoparticles

**DOI:** 10.3390/ma11060904

**Published:** 2018-05-28

**Authors:** Mahdi Ilka, Susanta Bera, Se-Hun Kwon

**Affiliations:** 1 School of Materials Science and Engineering, Pusan National University, Busan 46241, Korea; ilka@pusan.ac.kr (M.I.); berasusanta88@gmail.com (S.B.); 2 Global Frontier R&D Center for Hybrid Interface Materials, Pusan National University, Busan 46241, Korea; 3 Institute of Materials Technology, Pusan National University, Busan 46241, Korea

**Keywords:** low temperature solution method, SnO_2_ nanoparticles, surface defects, photoelectrochemical activity, photocatalytic activity

## Abstract

We report the successful synthesis of surface defective small size (SS) SnO_2_ nanoparticles (NPs) by adopting a low temperature surfactant free solution method. The structural properties of the NPs were analyzed using X-ray diffraction (XRD), field emission scanning electron microscopy (FESEM), and transmission electron microscopy (TEM). The presence of surface defects, especially oxygen vacancies, in the sample were characterized using micro-Raman spectroscopy, X-ray photoelectron spectroscopy (XPS), and photoluminescence emission. The Brunauer–Emmet–Teller (BET) nitrogen adsorption–desorption isotherms demonstrated the superior textural properties (high surface area and uniform pore size) of SS SnO_2_ compared to large size (LS) SnO_2_. A comparable study was drawn between SS SnO_2_ and LS SnO_2_ NPs and a significant decrease in the concentration of surface defects was observed for the LS sample. The results showed that surface defects significantly depend upon the size of the NPs. The surface defects formed within the band gap energy level of SnO_2_ significantly participated in the recombination process of photogenerated charge carriers, improving photochemical properties. Moreover, the SS SnO_2_ showed superior photoelectrochemical (PEC) and photocatalytic activities compared to the LS SnO_2_. The presence of a comparatively large number of surface defects due to its high surface area may enhance the photochemical activity by reducing the recombination rate of the photogenerated charges.

## 1. Introduction

The use of metal oxide semiconductors (MOS) in photochemical processes such as the photocatalytic degradation of water soluble organic pollutants and photocatalytic/photoelectrochemical water splitting has received great attention [1,2,3,4,5]. MOS, such as ZnO, TiO_2_, and SnO_2_, are well-known photocatalysts that can show efficient photocatalytic/photoelectrochemical activity through the extraction of photoexcited electrons and holes [1,3,4,5]. The functional oxide, SnO_2_, is a potential n-type semiconductor with outstanding optical, electrical, and electrochemical properties [6,7], and thus can exhibit efficient photocatalytic/photoelectrochemical activity [6,7,8]. However, this activity could be enhanced further by improving the separation of the photogenerated charges. 

Various extrinsic and intrinsic surface defects in semiconductors can act as active centers to trap photogenerated electrons and control the electron–hole recombination process [3,4,5,6,7,8]. Thus, surface defects can play a major role in the photogenerated charge separation process and enhance the photochemical process [3,4,5,6,7,8]. It is believed that the surface defects can vary with the size and shape of nanostructures, and in this respect, it has been found that small size SnO_2_ nanoparticles (NPs) exhibit more surface defects, such as oxygen vacancies, compared to SnO_2_ nanorods and nanospheres [6,7,9]. Also, their favorable textural properties have attracted considerable attention due to distinctive features such as high surface areas, uniform pore sizes and large pore volumes which allow efficient electrochemical and photochemical activities [10]. These features can have a strong effect on the adsorption and diffusion efficiency of guest molecules within the pore network, especially in photocatalysis [11]. Moreover, the properties can provide a regularly aligned pathway for the diffusion of molecules as well as offering a large number of active sites in the interconnected mesopore wall for interaction with guest molecules. To date, little emphasis has been placed on the ability of the size, shape, and surface defects of SnO_2_ nanoparticles to improve their photocatalytic/photoelectrochemical activity. Thus, it is suggested that small SnO_2_ nanoparticles with a high specific surface area will contain many surface defects, improving photochemical activity via prolonging the photogenerated charge recombination process. 

Generally, SnO_2_ NPs with surface defects can be prepared by hydrothermal and microwave syntheses [5,6,7,8]. However, these synthesis techniques require the use of stringent conditions to grow the surface defective SnO_2_ NPs. Moreover, it is very difficult to understand the mechanism (for a rational synthesis strategy) involved in hydrothermal processing [12]. In this respect, low temperature solution methods in air at atmospheric pressure are more advantageous than other approaches for the synthesis of surface defective SnO_2_ NPs for photochemical applications. In this work, we synthesized surface defective small size (SS) SnO_2_ NPs from a surfactant free precursor solution using a simple low temperature (95 °C) solution method in an air atmosphere. Structural (crystal phase, size, and morphology), surface defect, and textural (multi point BET surface area, porosity, and pore volume) analyses were used to characterize the material properties of the samples. Finally, the photochemical properties of the SS SnO_2_ NPs were systematically studied and compared with large size (LS) SnO_2_ NPs. The properties varied with NP size and greatly influenced the photoelectrochemical and photocatalytic activities of the NPs. 

## 2. Materials and Methods

### 2.1. Synthesis of Small Size (SS) and Large Size (LS) SnO_2_ NPs

Surface defective, small size (SS) SnO_2_ NPs were synthesized using a facile low temperature (95 °C) solution method using tin (II) 2-ethylhexanoate (TEH) (Sigma-Aldrich, Saint Louis, MO, USA 95%) in dimethyl formamide (DMF) solvent. In this synthesis, 1 g of TEH was thoroughly mixed with 200 mL DMF by continuous stirring. Subsequently, the aliquot was placed in an air oven at 95 °C for 24 h. The mixture was then centrifuged at 12,000 rpm for 10 min to separate the nanoparticles from the solution mixture. The product was washed several times using ethanol and deionized water separately. Finally, the samples were dried in an air oven (NEURONFIT, Seoul, Korea) at ~55 °C for 3 h, and SS SnO_2_ NPs were obtained. The calculated yield of SS SnO_2_ NPs was ~84%. To make large size (LS) SnO_2_ NPs, the SS NPs were heated at 800 °C for 2 h into a box furnace in air atmosphere.

### 2.2. Characterization of SnO_2_ NPs

The surface features, including the cluster sizes of the SnO_2_ samples, were analyzed using a field emission scanning electron microscope (FESEM, S-4800, Hitachi, Chiyoda-Ku, Japan). The microstructural properties of the SS SnO_2_ NPs were further characterized using a transmission electron microscope (TEM, JEM-2100F(HR), JEOL, Akishima, Japan) at an operating power of 200 kV. For the measurement, the samples were dispersed in methanol for 2 h, and the dispersed material was drop cast onto a carbon coated Cu grid (300 mesh). Subsequently, the grid was dried in an air atmosphere. An X-ray diffractometer (XRD, D8 ADVANCE, Bruker, Hong Kong, China) using Cu-*K_α_*_1_ at a wavelength of 1.5418 Å was used to analyze the crystallinity of the SnO_2_ samples. The surface areas of SS and LS SnO_2_ NPs were determined with Brunauer–Emmet–Teller (BET) nitrogen adsorption–desorption isotherms measurement at liquid nitrogen temperature (77 K) using a BET surface area and porosity analyzer (Autosorb-1, Quantachrome Instruments, Boynton Beach, FL, USA). Prior to the measurement, the samples were out-gassed in a vacuum at 90 °C for ~3 h. The pore size distribution was estimated by the Barrett–Joyner–Halenda (BJH) method and pore volume was determined from the amount of adsorbed nitrogen at P/P_0_ = 0.994225. The Raman spectra of the SnO_2_ samples were measured using a micro-Raman spectrometer (Xper Ram 200, Nano Base). The X-ray photoelectron spectra (XPS) of the SnO_2_ samples were measured using an X-ray photoelectron spectrometer (K-Alpha, Thermo Fisher Scientific, Waltham, MA, USA). Monochromated Al-*K_α_* was used as the X-ray source. The position of the C 1s peak was taken as a reference (binding energy, 284.6 eV). The absorption spectra of the samples were measured using the diffuse reflectance method with a UV-Vis-NIR spectrophotometer (UV3600, Shimadzu, Kyoto, Japan) with an ISR attachment. The room temperature photoluminescence emission spectra of the samples were recorded using a fluorescence spectrometer (LS-55, Perkin-Elmer, Waltham, MA, USA) with an excitation wavelength of 325 nm. 

### 2.3. Photoelectrochemical and Photocatalytic Activities 

Photoelectrochemical measurements of the SS and LS SnO_2_ samples were carried out using a modular high power potentiostat/galvanostat instrument (Autolab PGSTAT302N, Metrohm, Herisau, The Switzerland) with a standard three-electrode cell, under dark as well as visible light irradiation. A 50 W white LED lamp (FOCUS LED, AC 220 V, 6500 K, 4200 lm, Shanghai Haneup City Lighting Electric, Shanghai, China) was used as the visible light source. An Ag/AgCl (3 M KCl) electrode and a Pt foil were used as the reference electrode and counter electrode for the measurements, respectively. The working electrode was fabricated on cleaned FTO coated glass by the doctor-blading technique from a paste-like viscous material formed from the respective samples. The sample paste was produced by grinding the samples and mixing them with DMF solvent. The coated glass was then dried at 90 °C for ~4 h in the air oven. An aqueous solution of 0.1 M Na_2_SO_4_ was used as the electrolyte in this experiment. The photocurrent response was measured by manual control of on-off cycles. Electrochemical impedance spectra (EIS Nyquist plots) of the samples were measured in the frequency range of 0.01–100 KHz with an AC amplitude of 10 mV under visible light irradiation. Chronoamperometric study (photocurrent density vs. time, *I*–*t* curve) of the samples were performed at zero bias potential vs. Ag/AgCl (3 M KCl). 

The photocatalytic activity of the two samples during the degradation of an aqueous solution of methyl orange (MO) was measured under irradiation from the same visible light that was used in the photoelectrochemical measurements. Initially, 1.0 mg/mL of the photocatalyst was dispersed in 10^−6^ M of MO. The dispersed solutions were continuously stirred in the dark for 1 h to achieve an adsorption–desorption equilibrium. In this experiment, *C*_0_ and *C* indicate the initial concentration of the dye after achieving an adsorption–desorption equilibrium in the dark and the actual concentration of the MO dye solution after a specific light exposure time, respectively. The suspensions were centrifuged at different time intervals to measure their UV–Vis absorption spectra (UV1800, Shimadzu, Kyoto, Japan). The activity of the samples was determined from the plot of *C*/*C*_0_ against light irradiation time. 

## 3. Results and Discussion

### 3.1. Material Properties 

**Figure 1:**
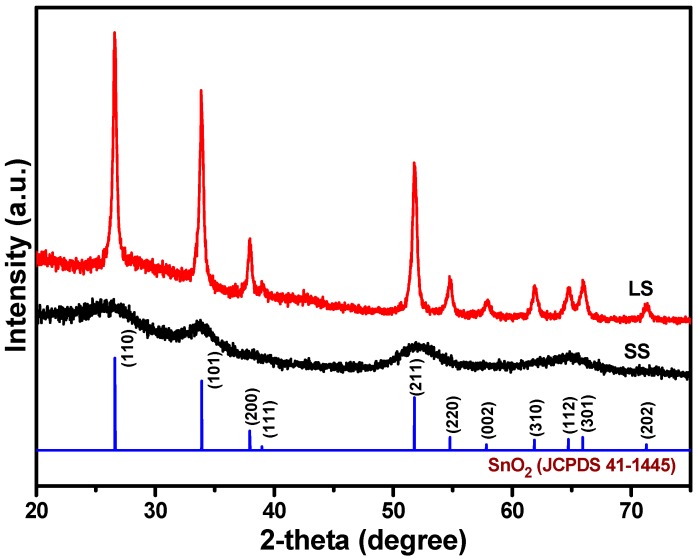
XRD patterns of the small size (SS) and large size (LS) SnO_2_ nanoparticles.

**Figure 2:**
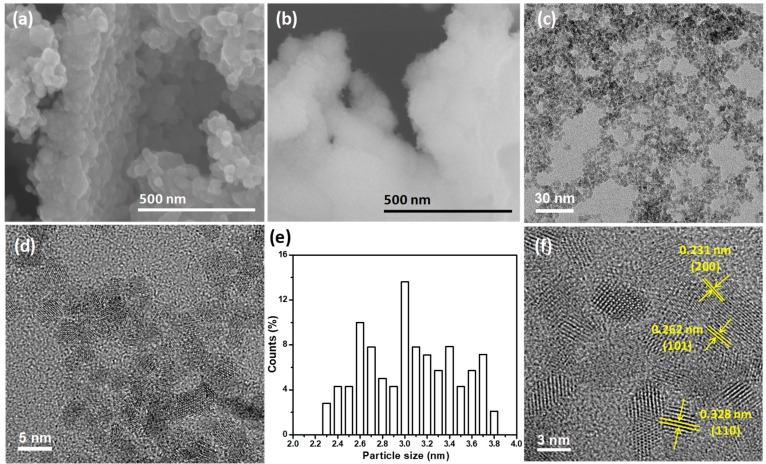
FESEM images of the LS (**a**) and SS (**b**) samples. TEM images (**c**,**d**), and HRTEM image (**f**) of the SS SnO_2_ nanoparticles (NPs). Particle size distribution (**e**) measured from (**c**).

In reaction medium, the majority of an Sn(II) precursor will form SnO_2_ NPs under an air atmosphere, while some of the Sn(II) ions will be introduced into the SnO_2_ lattice matrix during low temperature solution processing because of the comparable ionic radii of hexacoordinate Sn(IV) (0.69 Å) and Sn(II) (0.62 Å) [8]. Thus, the Sn(II) ions in the reaction medium can be doped into SnO_2_, resulting in the formation of oxygen vacancies [13]. Figure 1 shows the XRD patterns of the SS and LS SnO_2_ samples. The patterns were fully consistent with tetragonal SnO_2_ (JCPDS No. 41-1445). However, the SS sample showed broader diffraction peaks due to the small sizes of the SnO_2_ crystallites [14]. In contrast, the pattern of the LS sample showed sharp XRD peaks, demonstrating the significant crystalline growth of the SnO_2_ NPs, indicating the formation of a larger crystallite size. The average crystallite sizes of SS and LS samples were estimated to be 2.8 nm and 25.5 nm, respectively, using the Scherrer equation. A FESEM image (Figure 2a) of the LS confirmed the formation of larger SnO_2_ nanoclusters (20–50 nm). On the other hand, the FESEM image (Figure 2b) of the SS sample did not exhibit distinct grain boundaries, and the cluster/particle sizes of SnO_2_ could not be determined. Thus, it can be assumed that the particles of the SS sample would be very small. To determine the particle size, a TEM analysis of the SnO_2_ NPs was performed. The TEM images of the SS SnO_2_ sample are shown in Figure 2c,d,f. The particle size distribution curve (Figure 2e) of the SnO_2_ NPs showed a narrow size range (2.3–3.8 nm) for the NPs. The HRTEM images of the SS SnO_2_ NPs showed distinct lattice fringes with inter-planar distances of 0.328 nm, 0.262 nm and 0.231 nm, which corresponded to the (110), (101) and (200) planes of tetragonal SnO_2_, respectively, corroborating the XRD results. 

**Figure 3:**
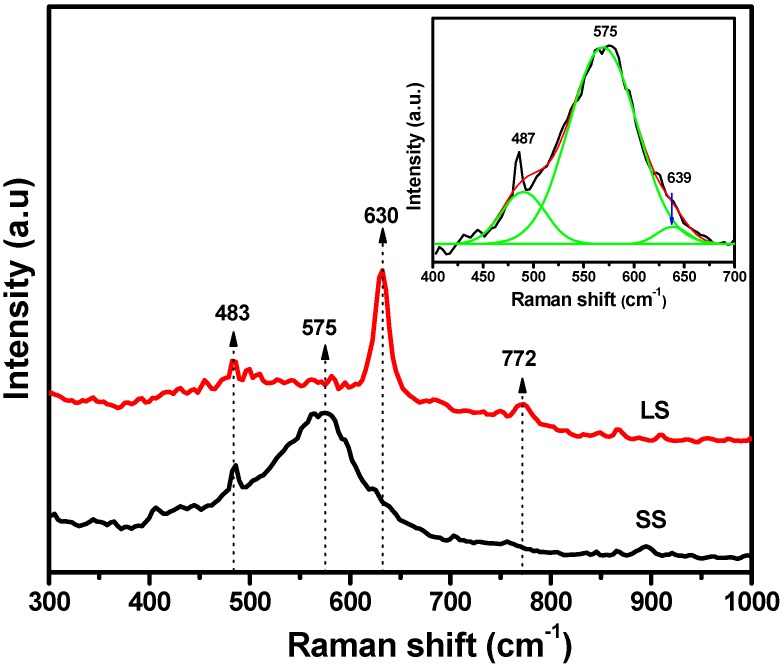
Raman spectra of the SS and LS samples; inset shows the magnified part of SS sample.

Raman spectroscopy is an important tool for the study of surface defects on nanoparticles. The Raman spectra (Figure 3) of the SS and LS SnO_2_ samples were measured to detect the presence of surface defects, such as oxygen vacancies, as well as the Raman active modes of tetragonal SnO_2_. The Raman spectrum of the LS sample contained three peaks at 483, 630 and 772 cm^−1^, which were attributed to the E_g_, A_1g_ and B_2g_ vibration modes, respectively, and were consistent with those of rutile bulk SnO_2_ [6,7]. However, an additional peak appeared at 575 cm^−1^ for the SS sample; this peak was attributed to surface defects on the SnO_2_ NPs [6]. This surface defect related peak was not detected in the LS sample. To understand the rutile bands, along with the surface related defect band on the SS sample, the broad peak was resolved into three Gaussian peaks (inset Figure 3). Additionally, a small shifting in the position of the Raman bands was noticed with respect to the LS sample. Kar et al. [7] also found the same observation for SnO_2_ nanoparticles compared to bulk SnO_2_. Therefore, surface defects, especially oxygen deficiencies, were found in the SS SnO_2_ NPs, indicating that the number of defect centers depends strongly on the size of the nanoparticles. 

**Figure 4:**
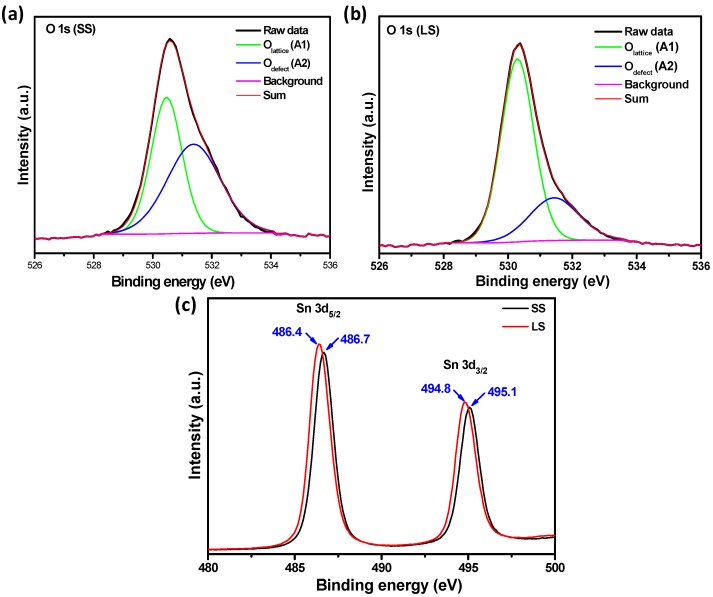
XPS data for the binding energies of (**a**) O 1s for the SS sample; (**b**) O 1s for the LS sample and (**c**) core levels of Sn 3d for both samples.

The presence of oxygen vacancies in the SnO_2_ samples was also confirmed by the XPS analysis. The XPS spectra of the SS and LS SnO_2_ samples are shown in Figure 4. The O 1s binding energy (Figure 4a) of the SS SnO_2_ sample was resolved into two Gaussian peaks with binding energies centered at 530.4 eV (A1) and 531.4 eV (A2), corresponding to lattice oxygen (O_lattice_) and the oxygen deficient region (O_defect_) in a tetragonal SnO_2_ crystal, respectively [5,8,15]. However, for the LS sample, the intensity of the oxygen defect related peak decreased (Figure 4b) [16,17]. The relative contents (R, calculated by dividing the area of the A2 peak by the area of the A1 peak) of oxygen defects in the SS and LS SnO_2_ samples were 1.13 and 0.37, respectively. This result clearly indicates a significant decrease in the amount of oxygen vacancies in the LS SnO_2_ NPs. The decrease of oxygen vacancies in the LS sample may have been induced from heating in the air atmosphere, and similar results were also found for ZnO [18,19]. The Sn binding energy peaks in the LS sample appeared at 486.4 eV and 494.8 eV, and were assigned as the Sn 3d_5/2_ and Sn 3d_3/2_ peaks of SnO_2_, respectively, as shown in Figure 4c [20]. However, the Sn3d peaks of the SS SnO_2_ sample were found at higher energy levels. As described above, a large number of oxygen vacancies acting as electron acceptors were present in the lattice of the SS SnO_2_. The presence of a greater number of electron acceptor centers near the Sn atoms may decrease the electron density of these atoms, resulting in an increase in the Sn3d binding energy [8,21]. This result indirectly demonstrated the existence of a much greater number of oxygen vacancies in the SS SnO_2_ sample than in the LS sample.

**Figure 5:**
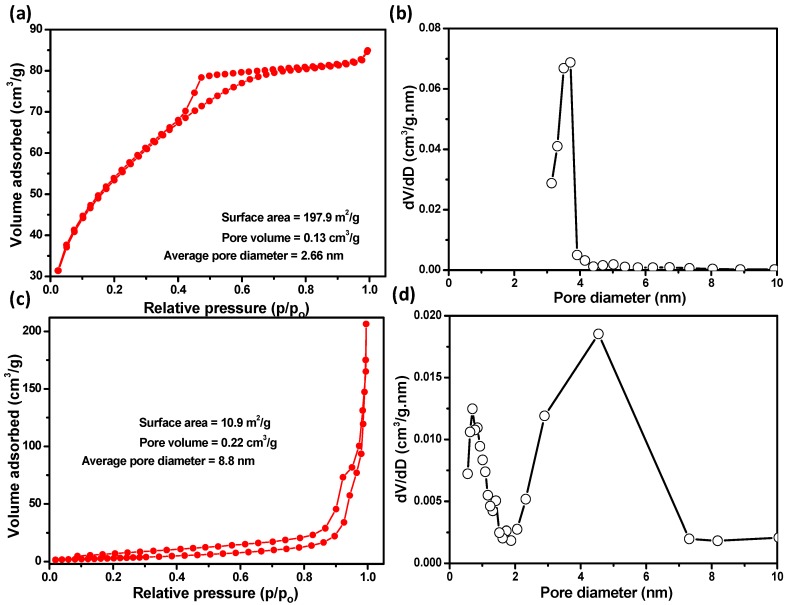
Multipoint BET nitrogen adsorption–desorption isotherms of (**a**) SS and (**c**) LS samples, and pore size distribution curves of (**b**) SS and (**d**) LS samples, calculated from the desorption branch of the isotherm.

The textural properties (Figure 5), i.e., the surface area, pore size, and pore volume, of the SS and LS samples were studied using multi-point BET nitrogen adsorption–desorption isotherm measurement. The isotherms of SS sample were type IV with an H4 hysteresis loop, indicating the presence of narrow, slit-like mesopores in the sample matrix [22] (Figure 5a,b). The measured average specific surface area, pore volume and pore size were 197.9 m^2^/g, 0.13 cm^3^/g and ~2.7 nm, respectively. The pore size distribution curves of the sample determined from the desorption branch showed a sharp peak at ~3.7 nm (Figure 5b). On the other hand, the LS sample was type IV with an H3 hysteresis loop, implying that it contained slit-shaped pores resulting from aggregates of plate-like particles [22] (Figure 5c,d). It was also noted that it exhibited bimodal wider pore size distribution. The specific surface area, pore volume and average pore size of the LS sample were 10.9 m^2^/g, 0.22 cm^3^/g and 8.8 nm, respectively. Therefore, the specific surface area of LS sample was found to be decreased by about 18 times. Moreover, the hysteresis loop, pore size and shape of mesopores of LS sample were found to be changed abruptly compared to the SS sample. The high specific surface area of the SS sample was comparatively higher than those of other surface defective SnO_2_ nanoparticles synthesized by various methods [5,7,23]. Thus, low temperature solution synthesis in an air atmosphere is advantageous for obtaining nanoparticles with a high specific surface area with uniform pore sizes which can play an important role in improving both their photoelectrochemical and photocatalytic activities.

### 3.2. Optical and Photochemical Properties 

**Figure 6:**
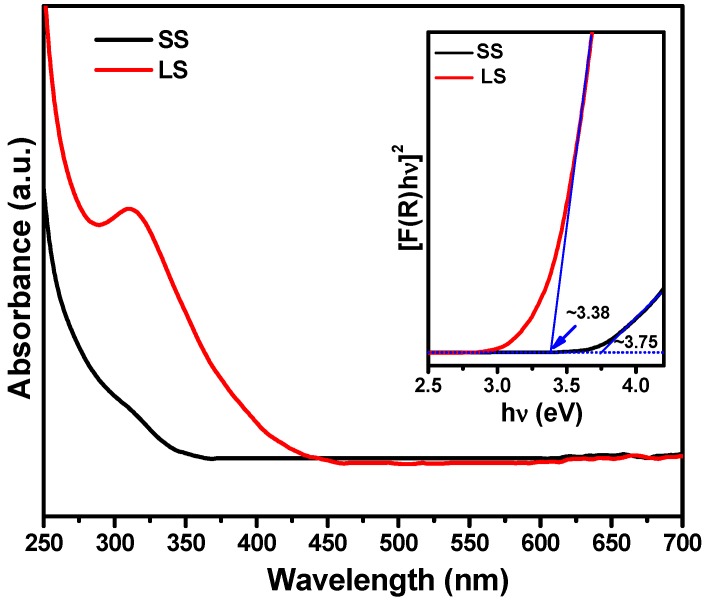
UV–Vis absorption spectra measured by the diffuse reflectance method. Inset shows an estimation of the band gap energy of the SS and LS samples.

**Figure 7:**
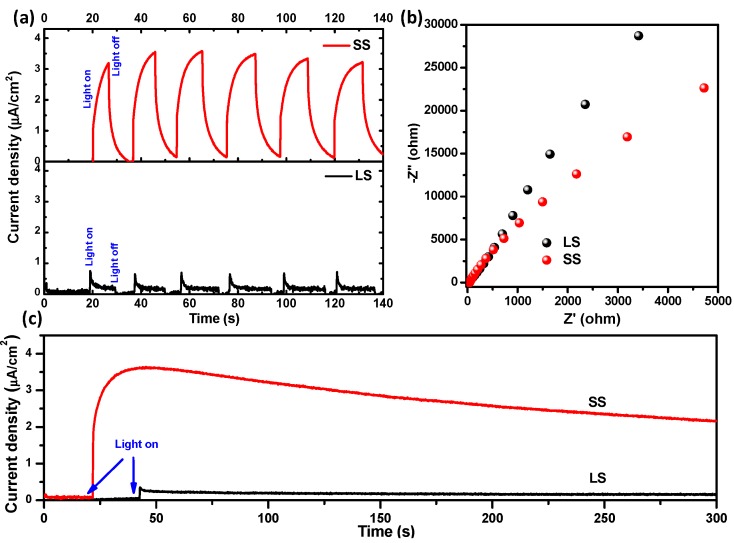
Photoelectrochemical properties of the SS and LS SnO_2_ samples: (**a**) photoresponse; (**b**) electrochemical impedance (EIS; Nyquist plots) of the electrodes measured under visible light irradiation; and (**c**) change in the current density with time (*I*–*t* curves) recorded under visible light illumination.

The UV–Vis absorbance spectra of the SS and LS SnO_2_ samples were measured by the diffuse reflectance method and are shown in Figure 6. Compared to the SS sample, the absorption edge of the LS sample showed a red shift. We estimated the optical direct band gap energy (inset of Figure 6) of SS sample to be ~3.75 eV, whereas the band gap energy of the LS sample was ~3.38 eV. The size dependence of the band gap energies of the SnO_2_ NPs was due to the quantum confinement effect [6]. The band gap was found to decrease from ~3.75 to ~3.38 eV, indicating that the SS SnO_2_ NPs synthesized at low temperatures had more discrete properties than the LS SnO_2_ NPs produced by further heating. It is well-known that the band gap energy of a material significantly affects its photochemical activity. In this work, although the SS SnO_2_ sample was found to have a higher band gap energy compared to the LS sample, it showed higher photochemical activity under visible light exposure than the LS sample (discussed later). A similar observation was reported by Anuchai et al. [5]; they concluded that the band gap energy has no direct influence on the efficiency of photocatalytic activity. In our study, the superior photochemical activity of SS sample could be due to the presence of a large number of surface defects and a high surface area, as confirmed from the Raman, XPS, and BET studies. The photoelectrochemical performances (Figure 7) of SS SnO_2_ and the LS sample were tested using a three-electrode system in a 0.1 M Na_2_SO_4_ solution. The working electrode was prepared by the doctor blade technique on FTO coated glass and acted as the photoanode for both the samples. The photocurrent responses of the samples were performed at 0 V vs. the Ag/AgCl (3 M KCl) reference electrode (Figure 7a). The SS sample showed enhanced photocurrent density compared to the LS sample, indicating a significant improvement in photogenerated charge transport and separation in the SS SnO_2_ [8,24,25]. The electrochemical impedance spectra (EIS) further confirmed the improvement in charge separation efficiency (Figure 7b). The arc radius of the EIS Nyquist plots was smaller for the SS SnO_2_ than for the LS SnO_2_. This result also suggests that the photogenerated charge separation was more efficient in the SS sample [8,24] which could be due to the larger surface area of the SS sample [26]. We also tested the photostability of the SS sample, and found that it showed significant PEC stability (Figure 7c) at 0 V vs. Ag/AgCl (3 M KCl).

**Figure 8:**
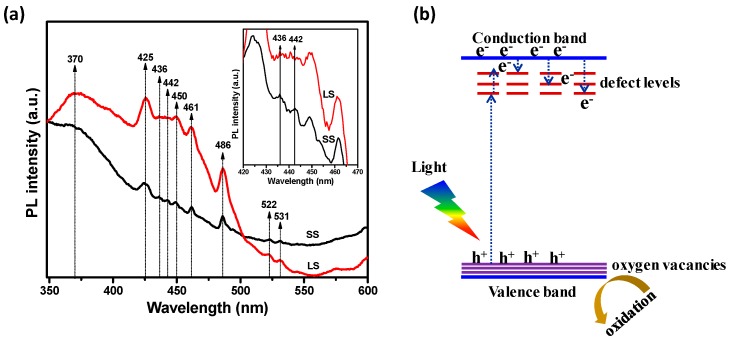
(**a**) Photoluminescence (PL) emission spectra of the SS and LS samples; inset shows the magnified part of the spectra; (**b**) schematic representation of the photochemical process of surface defective SnO_2_.

The improved photoelectrochemical properties of the SS SnO_2_ can be attributed to its enhanced charge transfer efficiency due to the influence of surface defects on the recombination process of the photoinduced electrons and holes. The photoluminescence (PL) spectra of the samples (Figure 8a) showed visible emissions at ~425 nm, ~436 nm, ~442 nm, ~450 nm, ~461 nm, ~486 nm, ~522 nm and ~531 nm, along with UV emission at ~370 nm [5,6,7,8,13]. The UV PL peak at ~370 nm originated from the band edge emission of SnO_2_ [6]. The visible emission from the SnO_2_ nanocrystals was associated with various intrinsic and extrinsic surface defects, such as oxygen vacancies and tin interstitials [5,6,7,8,13]. The formation of such defect levels in the SnO_2_ bandgap depends upon several factors, such as the preparation method and doping [5,6,8,13,27]. The UV and visible emissions of the samples are in competition with each other. Large, perfectly crystalline SnO_2_ nanocrystals show strong UV emission as well as a high ratio of UV emission to visible emission intensity [6,7]. In the present study, the LS SnO_2_ NPs exhibited stronger UV emission than the SS SnO_2_ NPs. It is therefore probable that the band edge emission of the SS SnO_2_ was restricted, and that excited electrons on the conduction level may have combined with the defect levels (oxygen vacancies and tin interstitials) within the bandgap energy, resulting in a decreased relative intensity ratio of UV to visible emission. The PL spectrum of the SS SnO_2_ also strongly justifies the above statement, as the visible emissions at ~436 nm and ~442 nm were more prominent than in the LS sample. Kasinathan et al. also found a prominent band centered at 442 nm in CeO_2_–TiO_2_, and they proposed the peak related to oxygen defects [28]. Thus, it can be surmised that the defect centers of the SS SnO_2_ NPs can strongly participate in the photoexcitation process and decrease the recombination rate. The Raman (Figure 3) and XPS (Figure 4) spectral studies confirmed the presence of surface defects, especially oxygen deficiencies, in the SS SnO_2_ sample. Thus, the surface oxygen defects can act as charge carrier traps, facilitating efficient charge separation and improving the photoelectrochemical properties (PEC) of the SS SnO_2_ sample [8,24]. A possible pathway for the separation of photogenerated electrons (e^−^) and holes (h^+^) is shown schematically in Figure 8b, where SnO_2_ absorbs photons of visible light to excite the electrons from its valence to surface defect levels, and then promotes the electrons to the higher levels to induce photochemical reactions [26]. Additionally, the textural properties of a material can improve its PEC properties. In this respect, the high specific surface area, and uniform pore sizes of the SS sample compared to the LS sample may allow faster diffusion of the electrolyte and thus, enhance redox kinetics and improve the PEC performance [10,11]. The applied bias photon-to-current efficiency (ABPE) was calculated for the photoelectrochemical processes of SS and LS samples at 0 V vs. Ag/AgCl (3 M KCl) using Equation (1). The ABPE of SS and LS samples were 0.00352% and 0.00057%, respectively.

ABPE (%) = [|J_photo_ × (|1.23 − V_appl._|)]/P_total_, (1)

where J_photo_ is the photocurrent density (mA/cm^2^), V_appl._ is the applied potential vs. RHE (reversible hydrogen electrode) and P_total_ is power density of incident light (mW/cm^2^).

The values were relatively low, but the SS sample exhibited an efficiency ~6 times higher than the LS sample. The enhanced photoelectrochemical efficiency of the SS sample in comparison to LS sample could be attributed to the efficient light absorption from the valence band to defect levels, efficient charge separation through defect centers within the band energy and transfer through the intraparticle diffusion path [29].

**Figure 9:**
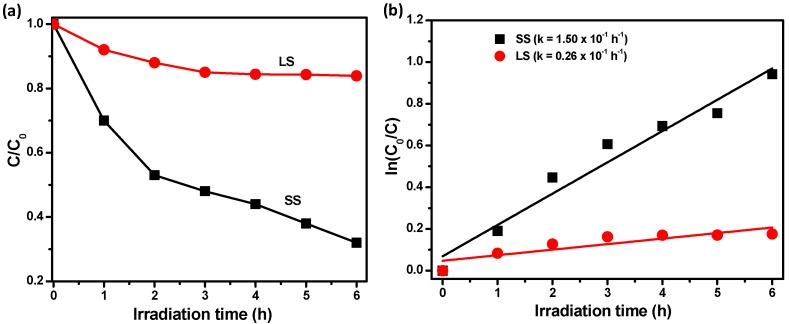
(**a**) Photocatalytic degradation of aqueous solutions of methyl orange dye in the presence of SS and LS SnO_2_ under UV–Vis light; (**b**) estimation of the pseudo-first order rate constants of the samples for the dye degradation; the rate constants for the degradation of the dye by the respective photocatalysts are embedded in the figure.

We also investigated the photocatalytic activity (Figure 9a) of the SnO_2_ samples towards the degradation of an aqueous solution of methyl orange (MO) under visible light irradiation. The SS SnO_2_ sample showed higher photocatalytic activity than the LS SnO_2_ sample. To quantify the photocatalytic efficiency, we calculated the rate constants of the degradation reaction using pseudo first order reaction kinetics (Figure 9b). The values of the degradation rate constant, k, were determined to be 1.50 × 10^−1^ and 0.26 × 10^−1^ h^−1^ for the SS and LS SnO_2_ samples, respectively. Therefore, the SS sample exhibited ~5.8 times greater photocatalytic activity than the LS sample. This could be attributed to the different textural properties and surface defect contents of the samples, as observed in the BET, Raman, XPS, and PL studies. Moreover, the large surface area and uniform pore sizes of the SS sample compared to the LS sample could offer a strong effect on the adsorption and diffusion efficiencies of dye molecules within the pore network during photocatalysis. However, the hybridization of noble nanometals or semiconductor nanoparticles with the SnO_2_ nanoparticles would be an efficient approach to further improve the photochemical activity of the material. 

## 4. Conclusions

A simple low temperature (95 °C) solution method for the synthesis of surface defective small size (SS) SnO_2_ nanoparticles (NPs) from a surfactant-free precursor solution in an air atmosphere was reported. Large size (LS) SnO_2_ NPs were prepared by further heating the SS NPs. The structural properties, textural properties, and the presence of surface defects, especially oxygen vacancies, of the SS and LS SnO_2_ NPs were analyzed. A significant decrease in the concentration of surface defects was observed for the LS sample. The photochemical properties varied upon changing the size and greatly influenced the photoelectrochemical and photocatalytic activities of the NPs. Moreover, the SS SnO_2_ showed enhanced photoelectrochemical (PEC) and photocatalytic activities compared to the LS SnO_2_. The comparatively large number of surface defects in the SS sample formed within the band gap energy level of SnO_2_ strongly participated in the photochemical process to reduce the recombination rate of the photogenerated electrons and holes.
